# Cariprazine add-on for resistant bipolar depression: preliminary results from an italian real-world experience

**DOI:** 10.1192/j.eurpsy.2023.623

**Published:** 2023-07-19

**Authors:** V. Martiadis, E. Pessina, A. Martini, F. Raffone, D. De Berardis

**Affiliations:** ^1^Department of Mental Health, ASL Napoli 1 Centro, Napoli; ^2^Department of Mental Health, ASL Cuneo 2, Cuneo; ^3^Department of Mental Health, ASL Teramo, Teramo, Italy

## Abstract

**Introduction:**

Depressive episodes represent the most frequent mood alteration in bipolar disorder (BD). Persistent depressive episodes and subsyndromic depressive symptoms often lead to poor quality of life and increase suicide risk. Recent studies have also shown that BD patients with a history of predominant depressive episodes generally show poorer response to pharmacological treatments. Although not specifically approved in Italy for use in bipolar depression, the scientific literature produced so far suggests the possible use of cariprazine in clinical conditions of bipolar depression that do not respond to conventional treatments.

**Objectives:**

The aim of the study was to evaluate, in a real-world multicentric Italian clinical setting, the efficacy and safety of cariprazine augmentation strategy in a sample of patients suffering from treatment-resistant bipolar depression.

**Methods:**

16 resistant bipolar depressed patients, whose resistance was defined according to The CINP Guidelines on the Definition and Evidence-Based Interventions for Treatment-Resistant Bipolar Disorder, were observed for 4 weeks after the add-on to previous mood stabilizing treatment of a cariprazine 1,5 mg fixed dose. Psychopathology at time 0 and at 1, 2, 3, and 4 weeks of treatment was evaluated using the Hamilton Depression Rating Scale, the Hamilton Anxiety Rating Scale, the Young Mania Rating Scale and the Brief Psychiatric Rating Scale; safety and tolerability of the therapy was measured by the UKU Side Effect Rating Scale.

**Results:**

Clinical improvement induced by 1,5 mg cariprazine add-on was effective and well tolerated in the study sample. Improvement in depression scores started from the first week, reaching about 35% reduction within 15 days and almost 50% in the following weeks (mean HDRS score from 24,7 to 13,2, GLM r.m. p<0,001); global psychopathology improved (mean BPRS score from 29,9 to 15,3 GLM r.m. p<0,001) as well as anxiety symptoms (mean HARS score from 26,5 to 16,5 GLM r.m. p=0,003). No manic shifts were observed during the observation period and the treatment was generally well tolerated.

**Conclusions:**

Despite the small number of patients examined and the short term of observation, cariprazine could represent an effective and safe enhancement strategy for patients with bipolar depression resistant to common pharmacological treatments. Further studies on larger samples are needed to confirm these preliminary findings. In addition, a more prolonged observation would be appropriate to highlight whether the beneficial effect of cariprazine add-on persists over time.

**Disclosure of Interest:**

None Declared

